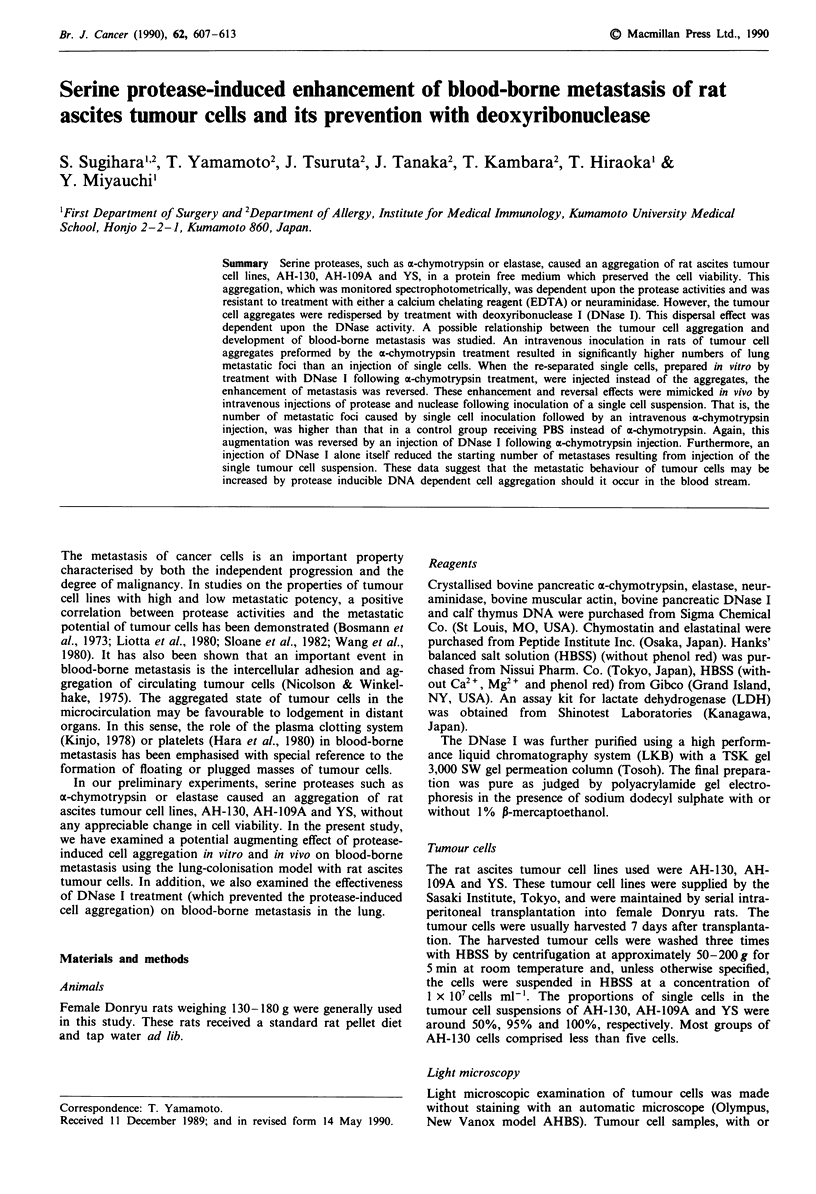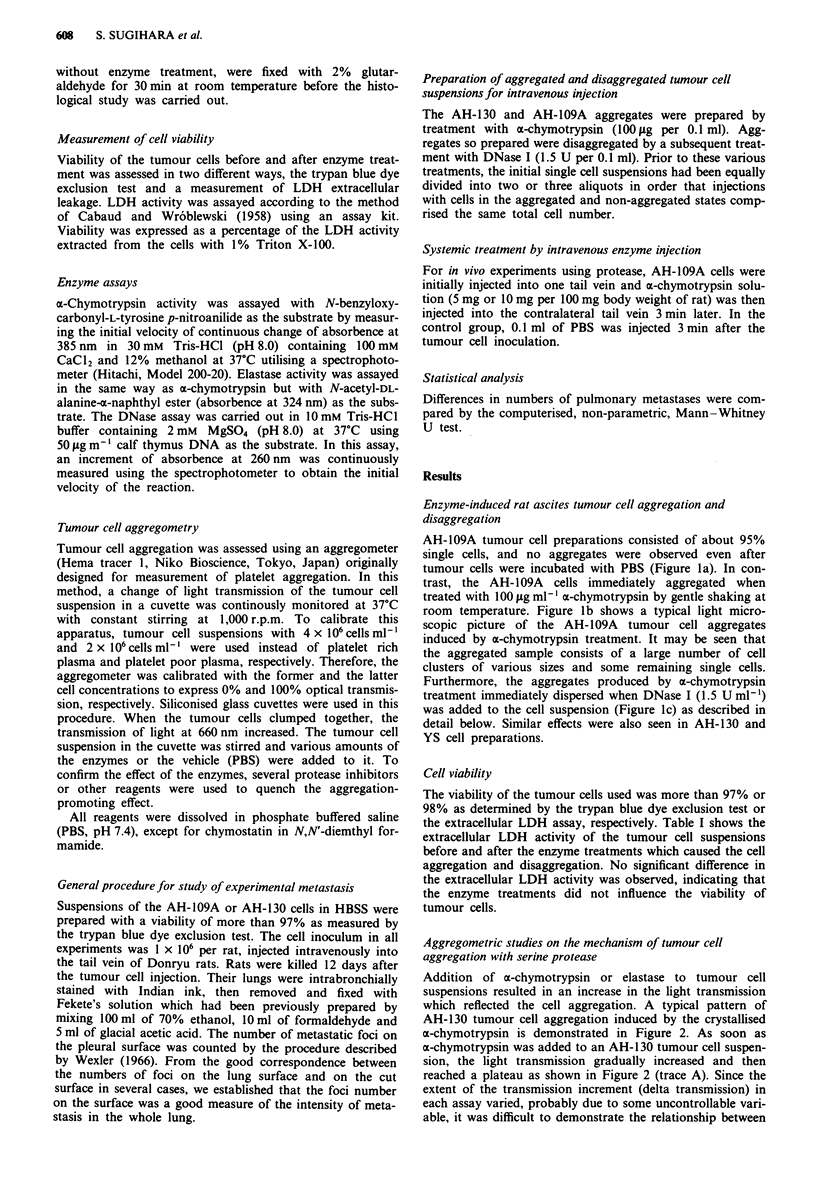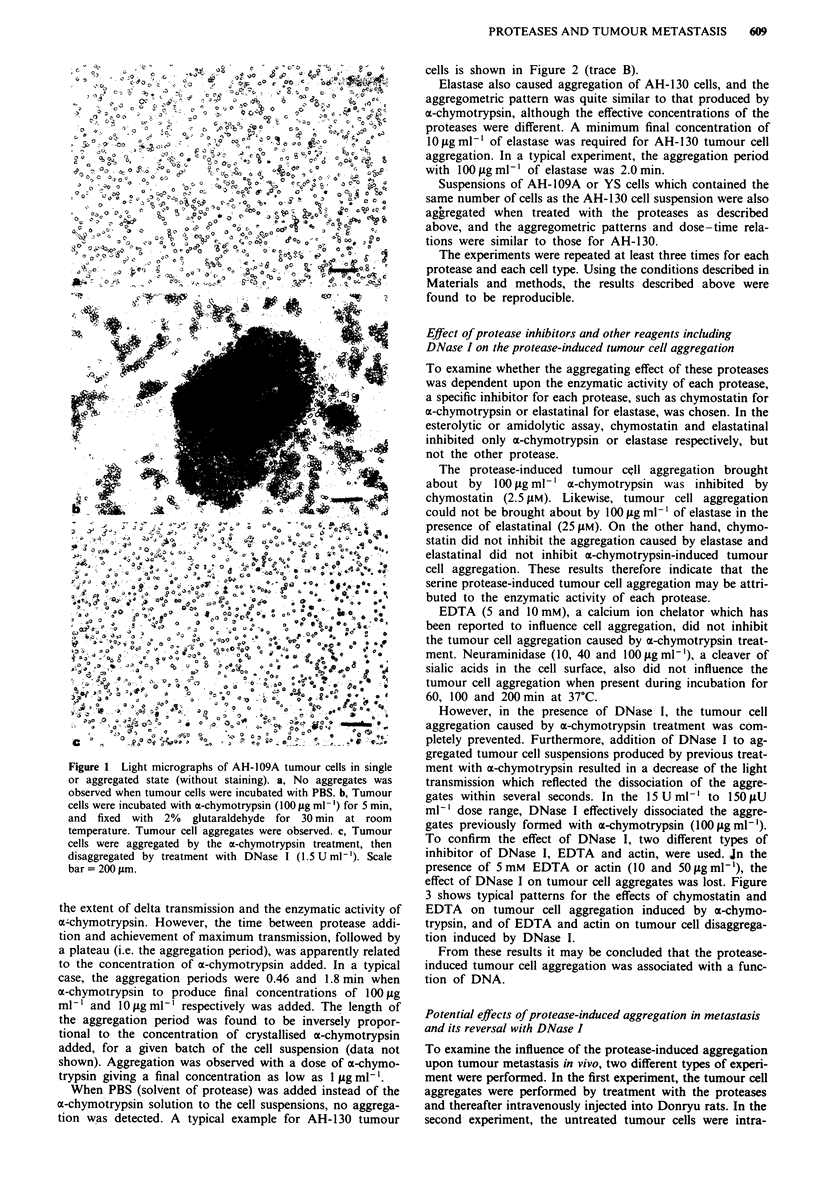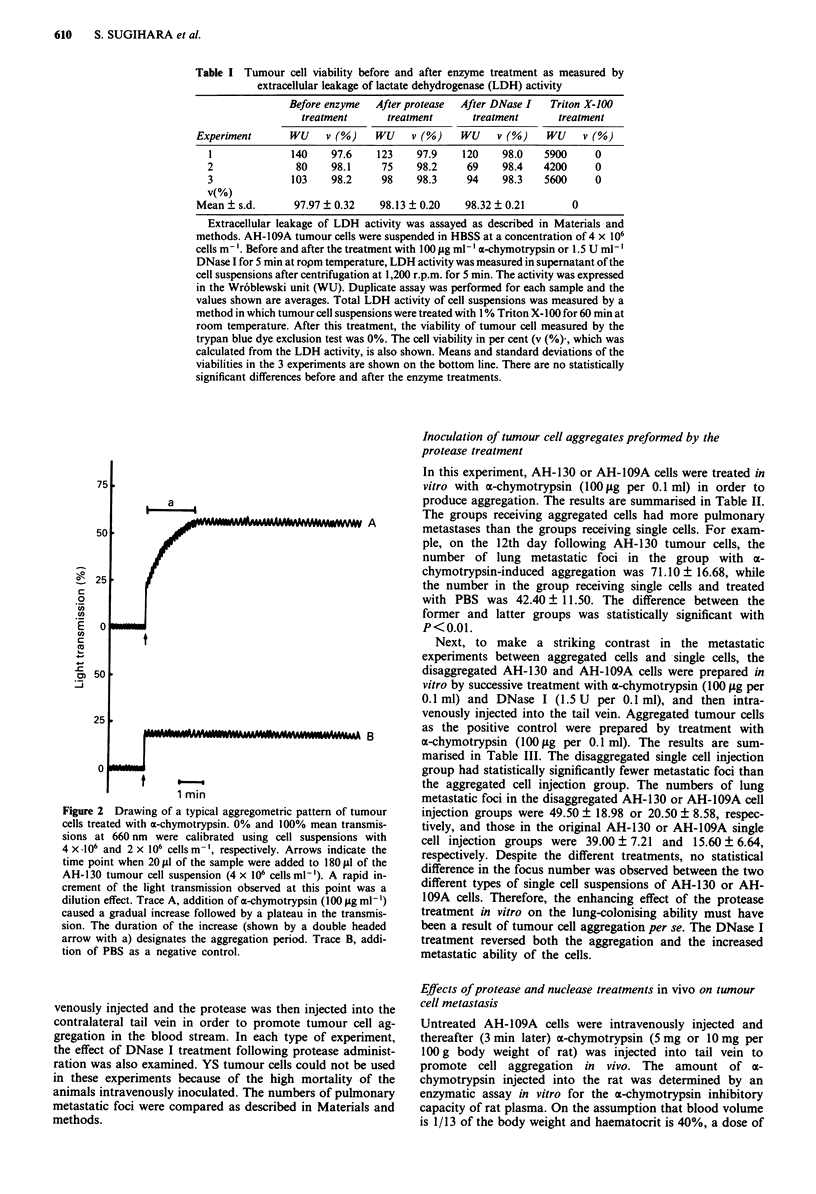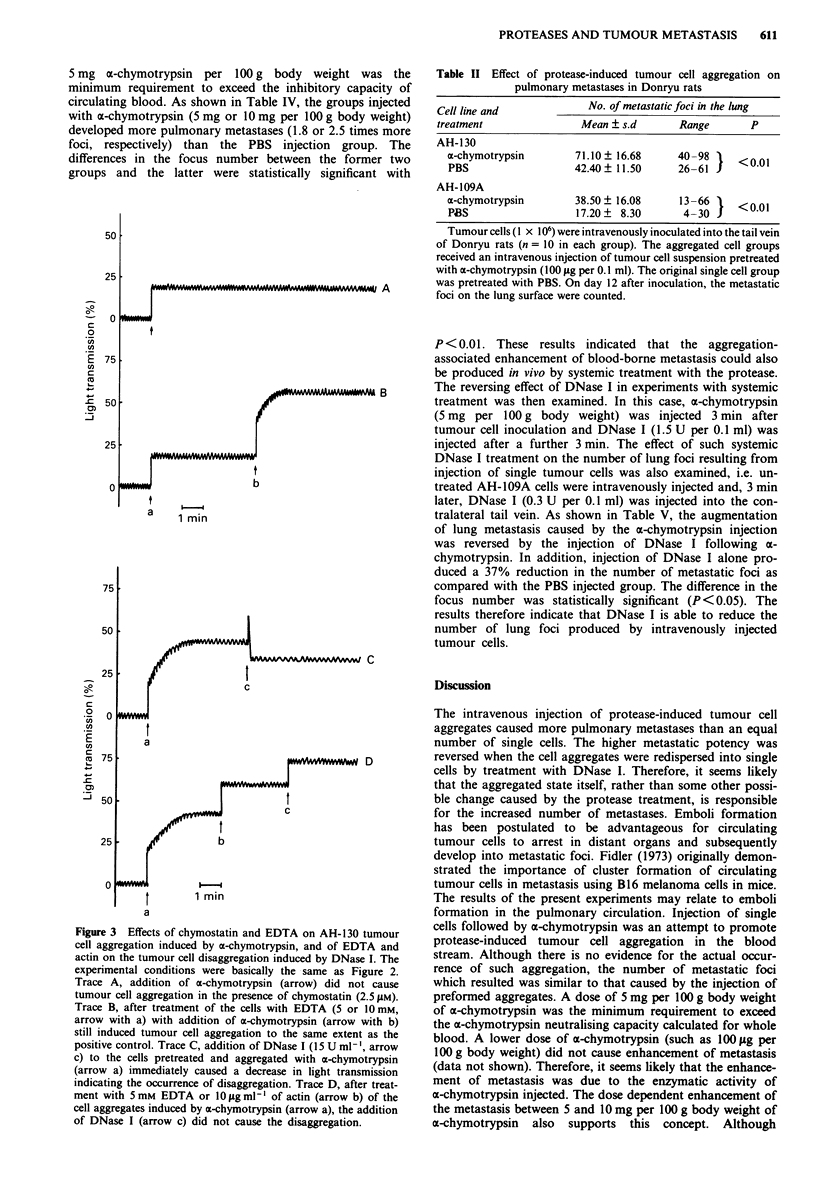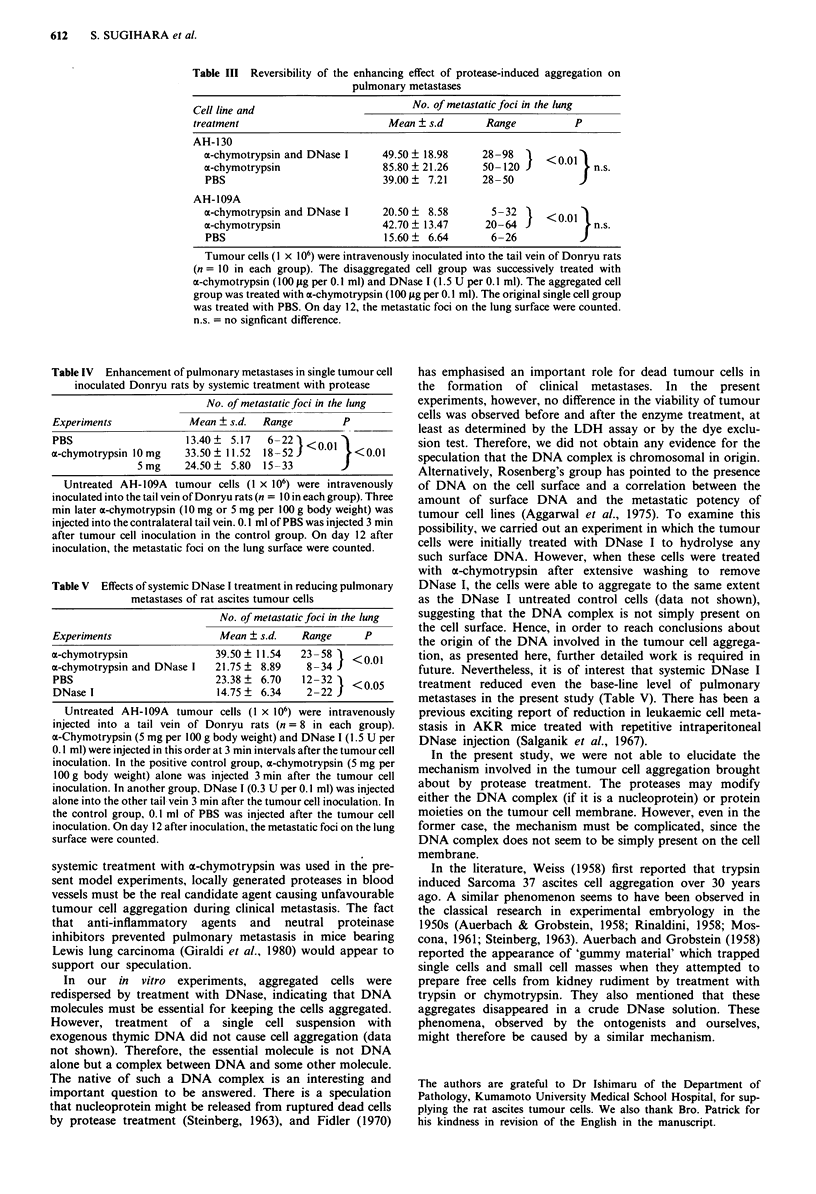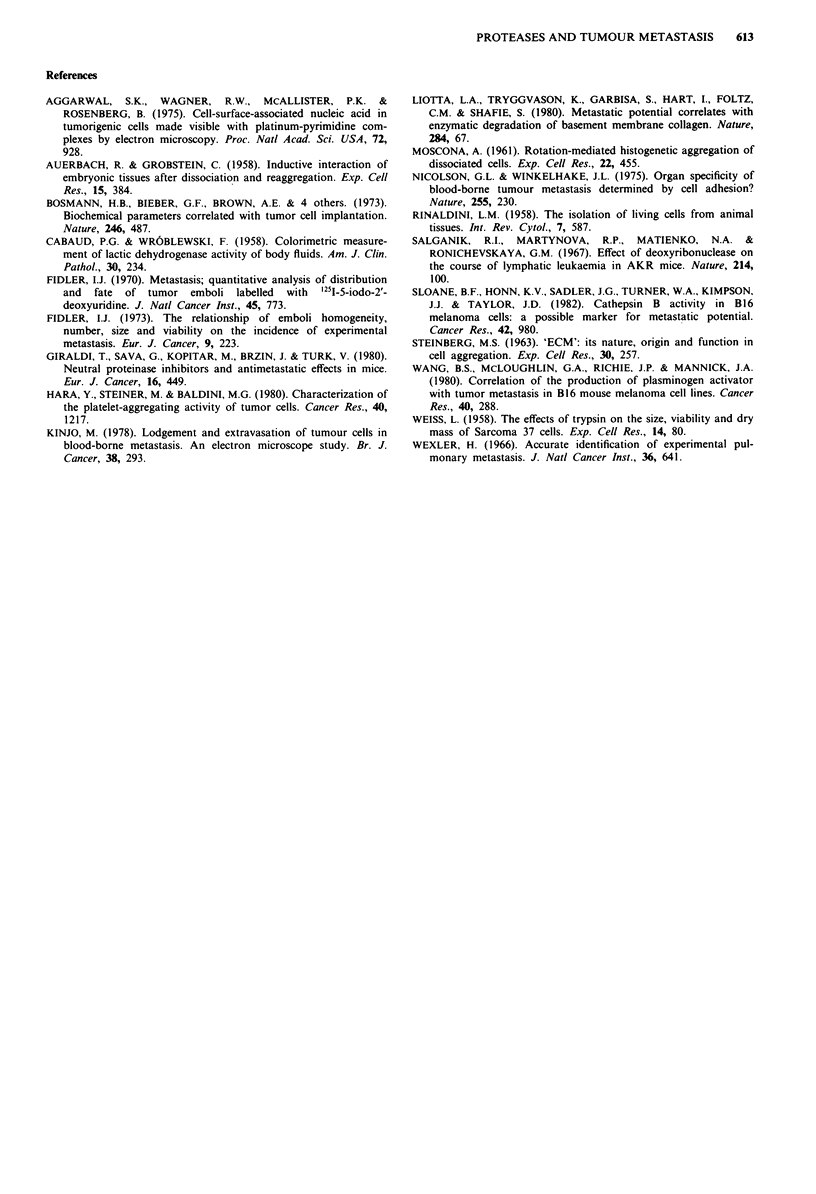# Serine protease-induced enhancement of blood-borne metastasis of rat ascites tumour cells and its prevention with deoxyribonuclease.

**DOI:** 10.1038/bjc.1990.339

**Published:** 1990-10

**Authors:** S. Sugihara, T. Yamamoto, J. Tsuruta, J. Tanaka, T. Kambara, T. Hiraoka, Y. Miyauchi

**Affiliations:** First Department of Surgery, Kumamoto University Medical School, Japan.

## Abstract

**Images:**


*Br. J. Cancer* (1990), **62**, 607–613 © Macmillan Press Ltd., 1990

### Serine protease-induced enhancement of blood-borne metastasis of rat ascites tumour cells and its prevention with deoxyribonuclease

S. Sugihara[1,2], T. Yamamoto[2], J. Tsuruta[2], J. Tanaka[2], T. Kambara[2], T. Hiraoka[1] & Y. Miyauchi[1]

[1]*First Department of Surgery and* [2]*Department of Allergy, Institute for Medical Immunology, Kumamoto University Medical School, Honjo 2-2-1, Kumamoto 860, Japan.*

**Summary** Serine proteases, such as α-chymotrypsin or elastase, caused an aggregation of rat ascites tumour cell lines, AH-130, AH-109A and YS, in a protein free medium which preserved the cell viability. This aggregation, which was monitored spectrophotometrically, was dependent upon the protease activities and was resistant to treatment with either a calcium chelating reagent (EDTA) or neuraminidase. However, the tumour cell aggregates were redispersed by treatment with deoxyribonuclease I (DNase I). This dispersal effect was dependent upon the DNase activity. A possible relationship between the tumour cell aggregation and development of blood-borne metastasis was studied. An intravenous inoculation in rats of tumour cell aggregates preformed by the α-chymotrypsin treatment resulted in significantly higher numbers of lung metastatic foci than an injection of single cells. When the re-separated single cells, prepared *in vitro* by treatment with DNase I following α-chymotrypsin treatment, were injected instead of the aggregates, the enhancement of metastasis was reversed. These enhancement and reversal effects were mimicked *in vivo* by intravenous injections of protease and nuclease following inoculation of a single cell suspension. That is, the number of metastatic foci caused by single cell inoculation followed by an intravenous α-chymotrypsin injection, was higher than that in a control group receiving PBS instead of α-chymotrypsin. Again, this augmentation was reversed by an injection of DNase I following α-chymotrypsin injection. Furthermore, an injection of DNase I alone itself reduced the starting number of metastases resulting from injection of the single tumour cell suspension. These data suggest that the metastatic behaviour of tumour cells may be increased by protease inducible DNA dependent cell aggregation should it occur in the blood stream.

The metastasis of cancer cells is an important property characterised by both the independent progression and the degree of malignancy. In studies on the properties of tumour cell lines with high and low metastatic potency, a positive correlation between protease activities and the metastatic potential of tumour cells has been demonstrated (Bosmann *et al.*, 1973; Liotta *et al.*, 1980; Sloane *et al.*, 1982; Wang *et al.*, 1980). It has also been shown that an important event in blood-borne metastasis is the intercellular adhesion and aggregation of circulating tumour cells (Nicolson & Winkelhake, 1975). The aggregated state of tumour cells in the microcirculation may be favourable to lodgement in distant organs. In this sense, the role of the plasma clotting system (Kinjo, 1978) or platelets (Hara *et al.*, 1980) in blood-borne metastasis has been emphasised with special reference to the formation of floating or plugged masses of tumour cells.

In our preliminary experiments, serine proteases such as α-chymotrypsin or elastase caused an aggregation of rat ascites tumour cell lines, AH-130, AH-109A and YS, without any appreciable change in cell viability. In the present study, we have examined a potential augmenting effect of protease-induced cell aggregation *in vitro* and *in vivo* on blood-borne metastasis using the lung-colonisation model with rat ascites tumour cells. In addition, we also examined the effectiveness of DNase I treatment (which prevented the protease-induced cell aggregation) on blood-borne metastasis in the lung.

### Materials and methods

### Animals

Female Donryu rats weighing 130–180 g were generally used in this study. These rats received a standard rat pellet diet and tap water *ad lib.*

Correspondence: T. Yamamoto.
Received 11 December 1989; and in revised form 14 May 1990.

### Reagents

Crystallised bovine pancreatic α-chymotrypsin, elastase, neuraminidase, bovine muscular actin, bovine pancreatic DNase I and calf thymus DNA were purchased from Sigma Chemical Co. (St Louis, MO, USA). Chymostatin and elastatinal were purchased from Peptide Institute Inc. (Osaka, Japan). Hanks' balanced salt solution (HBSS) (without phenol red) was purchased from Nissui Pharm. Co. (Tokyo, Japan), HBSS (without $Ca^{2+}$, $Mg^{2+}$ and phenol red) from Gibco (Grand Island, NY, USA). An assay kit for lactate dehydrogenase (LDH) was obtained from Shinotest Laboratories (Kanagawa, Japan).

The DNase I was further purified using a high performance liquid chromatography system (LKB) with a TSK gel 3,000 SW gel permeation column (Tosoh). The final preparation was pure as judged by polyacrylamide gel electrophoresis in the presence of sodium dodecyl sulphate with or without 1% β-mercaptoethanol.

### Tumour cells

The rat ascites tumour cell lines used were AH-130, AH-109A and YS. These tumour cell lines were supplied by the Sasaki Institute, Tokyo, and were maintained by serial intraperitoneal transplantation into female Donryu rats. The tumour cells were usually harvested 7 days after transplantation. The harvested tumour cells were washed three times with HBSS by centrifugation at approximately 50–200 *g* for 5 min at room temperature and, unless otherwise specified, the cells were suspended in HBSS at a concentration of $1 \times 10^7$ cells $ml^{-1}$. The proportions of single cells in the tumour cell suspensions of AH-130, AH-109A and YS were around 50%, 95% and 100%, respectively. Most groups of AH-130 cells comprised less than five cells.

### Light microscopy

Light microscopic examination of tumour cells was made without staining with an automatic microscope (Olympus, New Vanox model AHBS). Tumour cell samples, with or without enzyme treatment, were fixed with 2% glutaraldehyde for 30 min at room temperature before the histological study was carried out.

*Measurement of cell viability*

Viability of the tumour cells before and after enzyme treatment was assessed in two different ways, the trypan blue dye exclusion test and a measurement of LDH extracellular leakage. LDH activity was assayed according to the method of Cabaud and Wróblewski (1958) using an assay kit. Viability was expressed as a percentage of the LDH activity extracted from the cells with 1% Triton X-100.

*Enzyme assays*

α-Chymotrypsin activity was assayed with *N*-benzyloxycarbonyl-L-tyrosine *p*-nitroanilide as the substrate by measuring the initial velocity of continuous change of absorbence at 385 nm in 30 mM Tris-HCl (pH 8.0) containing 100 mM $CaCl_2$ and 12% methanol at 37°C utilising a spectrophotometer (Hitachi, Model 200-20). Elastase activity was assayed in the same way as α-chymotrypsin but with *N*-acetyl-DL-alanine-α-naphthyl ester (absorbence at 324 nm) as the substrate. The DNase assay was carried out in 10 mM Tris-HCl buffer containing 2 mM $MgSO_4$ (pH 8.0) at 37°C using $50\ \mu g\ m^{-1}$ calf thymus DNA as the substrate. In this assay, an increment of absorbence at 260 nm was continuously measured using the spectrophotometer to obtain the initial velocity of the reaction.

*Tumour cell aggregometry*

Tumour cell aggregation was assessed using an aggregometer (Hema tracer 1, Niko Bioscience, Tokyo, Japan) originally designed for measurement of platelet aggregation. In this method, a change of light transmission of the tumour cell suspension in a cuvette was continously monitored at 37°C with constant stirring at 1,000 r.p.m. To calibrate this apparatus, tumour cell suspensions with $4 \times 10^6$ cells $ml^{-1}$ and $2 \times 10^6$ cells $ml^{-1}$ were used instead of platelet rich plasma and platelet poor plasma, respectively. Therefore, the aggregometer was calibrated with the former and the latter cell concentrations to express 0% and 100% optical transmission, respectively. Siliconised glass cuvettes were used in this procedure. When the tumour cells clumped together, the transmission of light at 660 nm increased. The tumour cell suspension in the cuvette was stirred and various amounts of the enzymes or the vehicle (PBS) were added to it. To confirm the effect of the enzymes, several protease inhibitors or other reagents were used to quench the aggregation-promoting effect.

All reagents were dissolved in phosphate buffered saline (PBS, pH 7.4), except for chymostatin in *N,N'*-diemthyl formamide.

*General procedure for study of experimental metastasis*

Suspensions of the AH-109A or AH-130 cells in HBSS were prepared with a viability of more than 97% as measured by the trypan blue dye exclusion test. The cell inoculum in all experiments was $1 \times 10^6$ per rat, injected intravenously into the tail vein of Donryu rats. Rats were killed 12 days after the tumour cell injection. Their lungs were intrabronchially stained with Indian ink, then removed and fixed with Fekete's solution which had been previously prepared by mixing 100 ml of 70% ethanol, 10 ml of formaldehyde and 5 ml of glacial acetic acid. The number of metastatic foci on the pleural surface was counted by the procedure described by Wexler (1966). From the good correspondence between the numbers of foci on the lung surface and on the cut surface in several cases, we established that the foci number on the surface was a good measure of the intensity of metastasis in the whole lung.

*Preparation of aggregated and disaggregated tumour cell suspensions for intravenous injection*

The AH-130 and AH-109A aggregates were prepared by treatment with α-chymotrypsin (100 μg per 0.1 ml). Aggregates so prepared were disaggregated by a subsequent treatment with DNase I (1.5 U per 0.1 ml). Prior to these various treatments, the initial single cell suspensions had been equally divided into two or three aliquots in order that injections with cells in the aggregated and non-aggregated states comprised the same total cell number.

*Systemic treatment by intravenous enzyme injection*

For *in vivo* experiments using protease, AH-109A cells were initially injected into one tail vein and α-chymotrypsin solution (5 mg or 10 mg per 100 mg body weight of rat) was then injected into the contralateral tail vein 3 min later. In the control group, 0.1 ml of PBS was injected 3 min after the tumour cell inoculation.

*Statistical analysis*

Differences in numbers of pulmonary metastases were compared by the computerised, non-parametric, Mann–Whitney U test.

### Results

*Enzyme-induced rat ascites tumour cell aggregation and disaggregation*

AH-109A tumour cell preparations consisted of about 95% single cells, and no aggregates were observed even after tumour cells were incubated with PBS (Figure 1a). In contrast, the AH-109A cells immediately aggregated when treated with $100\ \mu g\ ml^{-1}$ α-chymotrypsin by gentle shaking at room temperature. Figure 1b shows a typical light microscopic picture of the AH-109A tumour cell aggregates induced by α-chymotrypsin treatment. It may be seen that the aggregated sample consists of a large number of cell clusters of various sizes and some remaining single cells. Furthermore, the aggregates produced by α-chymotrypsin treatment immediately dispersed when DNase I ($1.5\ U\ ml^{-1}$) was added to the cell suspension (Figure 1c) as described in detail below. Similar effects were also seen in AH-130 and YS cell preparations.

*Cell viability*

The viability of the tumour cells used was more than 97% or 98% as determined by the trypan blue dye exclusion test or the extracellular LDH assay, respectively. Table I shows the extracellular LDH activity of the tumour cell suspensions before and after the enzyme treatments which caused the cell aggregation and disaggregation. No significant difference in the extracellular LDH activity was observed, indicating that the enzyme treatments did not influence the viability of tumour cells.

*Aggregometric studies on the mechanism of tumour cell aggregation with serine protease*

Addition of α-chymotrypsin or elastase to tumour cell suspensions resulted in an increase in the light transmission which reflected the cell aggregation. A typical pattern of AH-130 tumour cell aggregation induced by the crystallised α-chymotrypsin is demonstrated in Figure 2. As soon as α-chymotrypsin was added to an AH-130 tumour cell suspension, the light transmission gradually increased and then reached a plateau as shown in Figure 2 (trace A). Since the extent of the transmission increment (delta transmission) in each assay varied, probably due to some uncontrollable variable, it was difficult to demonstrate the relationship between the extent of delta transmission and the enzymatic activity of α-chymotrypsin. However, the time between protease addition and achievement of maximum transmission, followed by a plateau (i.e. the aggregation period), was apparently related to the concentration of α-chymotrypsin added. In a typical case, the aggregation periods were 0.46 and 1.8 min when α-chymotrypsin to produce final concentrations of 100 μg ml$^{-1}$ and 10 μg ml$^{-1}$ respectively was added. The length of the aggregation period was found to be inversely proportional to the concentration of crystallised α-chymotrypsin added, for a given batch of the cell suspension (data not shown). Aggregation was observed with a dose of α-chymotrypsin giving a final concentration as low as 1 μg ml$^{-1}$.

When PBS (solvent of protease) was added instead of the α-chymotrypsin solution to the cell suspensions, no aggregation was detected. A typical example for AH-130 tumour cells is shown in Figure 2 (trace B).

Elastase also caused aggregation of AH-130 cells, and the aggregometric pattern was quite similar to that produced by α-chymotrypsin, although the effective concentrations of the proteases were different. A minimum final concentration of 10 μg ml$^{-1}$ of elastase was required for AH-130 tumour cell aggregation. In a typical experiment, the aggregation period with 100 μg ml$^{-1}$ of elastase was 2.0 min.

Suspensions of AH-109A or YS cells which contained the same number of cells as the AH-130 cell suspension were also aggregated when treated with the proteases as described above, and the aggregometric patterns and dose–time relations were similar to those for AH-130.

The experiments were repeated at least three times for each protease and each cell type. Using the conditions described in Materials and methods, the results described above were found to be reproducible.

### *Effect of protease inhibitors and other reagents including DNase I on the protease-induced tumour cell aggregation*

To examine whether the aggregating effect of these proteases was dependent upon the enzymatic activity of each protease, a specific inhibitor for each protease, such as chymostatin for α-chymotrypsin or elastatinal for elastase, was chosen. In the esterolytic or amidolytic assay, chymostatin and elastatinal inhibited only α-chymotrypsin or elastase respectively, but not the other protease.

The protease-induced tumour cell aggregation brought about by 100 μg ml$^{-1}$ α-chymotrypsin was inhibited by chymostatin (2.5 μM). Likewise, tumour cell aggregation could not be brought about by 100 μg ml$^{-1}$ of elastase in the presence of elastatinal (25 μM). On the other hand, chymostatin did not inhibit the aggregation caused by elastase and elastatinal did not inhibit α-chymotrypsin-induced tumour cell aggregation. These results therefore indicate that the serine protease-induced tumour cell aggregation may be attributed to the enzymatic activity of each protease.

EDTA (5 and 10 mM), a calcium ion chelator which has been reported to influence cell aggregation, did not inhibit the tumour cell aggregation caused by α-chymotrypsin treatment. Neuraminidase (10, 40 and 100 μg ml$^{-1}$), a cleaver of sialic acids in the cell surface, also did not influence the tumour cell aggregation when present during incubation for 60, 100 and 200 min at 37°C.

However, in the presence of DNase I, the tumour cell aggregation caused by α-chymotrypsin treatment was completely prevented. Furthermore, addition of DNase I to aggregated tumour cell suspensions produced by previous treatment with α-chymotrypsin resulted in a decrease of the light transmission which reflected the dissociation of the aggregates within several seconds. In the 15 U ml$^{-1}$ to 150 μU ml$^{-1}$ dose range, DNase I effectively dissociated the aggregates previously formed with α-chymotrypsin (100 μg ml$^{-1}$). To confirm the effect of DNase I, two different types of inhibitor of DNase I, EDTA and actin, were used. In the presence of 5 mM EDTA or actin (10 and 50 μg ml$^{-1}$), the effect of DNase I on tumour cell aggregates was lost. Figure 3 shows typical patterns for the effects of chymostatin and EDTA on tumour cell aggregation induced by α-chymotrypsin, and of EDTA and actin on tumour cell disaggregation induced by DNase I.

From these results it may be concluded that the protease-induced tumour cell aggregation was associated with a function of DNA.

### *Potential effects of protease-induced aggregation in metastasis and its reversal with DNase I*

To examine the influence of the protease-induced aggregation upon tumour metastasis *in vivo*, two different types of experiment were performed. In the first experiment, the tumour cell aggregates were performed by treatment with the proteases and thereafter intravenously injected into Donryu rats. In the second experiment, the untreated tumour cells were intra-

**Table I** Tumour cell viability before and after enzyme treatment as measured by extracellular leakage of lactate dehydrogenase (LDH) activity

| Experiment | *Before enzyme treatment* | | *After protease treatment* | | *After DNase I treatment* | | *Triton X-100 treatment* | |
|---|---|---|---|---|---|---|---|---|
| | *WU* | *v (%)* | *WU* | *v (%)* | *WU* | *v (%)* | *WU* | *v (%)* |
| 1 | 140 | 97.6 | 123 | 97.9 | 120 | 98.0 | 5900 | 0 |
| 2 | 80 | 98.1 | 75 | 98.2 | 69 | 98.4 | 4200 | 0 |
| 3 | 103 | 98.2 | 98 | 98.3 | 94 | 98.3 | 5600 | 0 |
| v(%) Mean ± s.d. | 97.97 ± 0.32 | | 98.13 ± 0.20 | | 98.32 ± 0.21 | | 0 | |

Extracellular leakage of LDH activity was assayed as described in Materials and methods. AH-109A tumour cells were suspended in HBSS at a concentration of $4 \times 10^6$ cells $m^{-1}$. Before and after the treatment with 100 μg $ml^{-1}$ α-chymotrypsin or 1.5 U $ml^{-1}$ DNase I for 5 min at ropm temperature, LDH activity was measured in supernatant of the cell suspensions after centrifugation at 1,200 r.p.m. for 5 min. The activity was expressed in the Wróblewski unit (WU). Duplicate assay was performed for each sample and the values shown are averages. Total LDH activity of cell suspensions was measured by a method in which tumour cell suspensions were treated with 1% Triton X-100 for 60 min at room temperature. After this treatment, the viability of tumour cell measured by the trypan blue dye exclusion test was 0%. The cell viability in per cent (v (%)·, which was calculated from the LDH activity, is also shown. Means and standard deviations of the viabilities in the 3 experiments are shown on the bottom line. There are no statistically significant differences before and after the enzyme treatments.

**Figure 2** Drawing of a typical aggregometric pattern of tumour cells treated with α-chymotrypsin. 0% and 100% mean transmissions at 660 nm were calibrated using cell suspensions with $4 \times 10^6$ and $2 \times 10^6$ cells $m^{-1}$, respectively. Arrows indicate the time point when 20 μl of the sample were added to 180 μl of the AH-130 tumour cell suspension ($4 \times 10^6$ cells $ml^{-1}$). A rapid increment of the light transmission observed at this point was a dilution effect. Trace A, addition of α-chymotrypsin (100 μg $ml^{-1}$) caused a gradual increase followed by a plateau in the transmission. The duration of the increase (shown by a double headed arrow with a) designates the aggregation period. Trace B, addition of PBS as a negative control.

venously injected and the protease was then injected into the contralateral tail vein in order to promote tumour cell aggregation in the blood stream. In each type of experiment, the effect of DNase I treatment following protease administration was also examined. YS tumour cells could not be used in these experiments because of the high mortality of the animals intravenously inoculated. The numbers of pulmonary metastatic foci were compared as described in Materials and methods.

*Inoculation of tumour cell aggregates preformed by the protease treatment*

In this experiment, AH-130 or AH-109A cells were treated *in vitro* with α-chymotrypsin (100 μg per 0.1 ml) in order to produce aggregation. The results are summarised in Table II. The groups receiving aggregated cells had more pulmonary metastases than the groups receiving single cells. For example, on the 12th day following AH-130 tumour cells, the number of lung metastatic foci in the group with α-chymotrypsin-induced aggregation was 71.10 ± 16.68, while the number in the group receiving single cells and treated with PBS was 42.40 ± 11.50. The difference between the former and latter groups was statistically significant with $P<0.01$.

Next, to make a striking contrast in the metastatic experiments between aggregated cells and single cells, the disaggregated AH-130 and AH-109A cells were prepared *in vitro* by successive treatment with α-chymotrypsin (100 μg per 0.1 ml) and DNase I (1.5 U per 0.1 ml), and then intravenously injected into the tail vein. Aggregated tumour cells as the positive control were prepared by treatment with α-chymotrypsin (100 μg per 0.1 ml). The results are summarised in Table III. The disaggregated single cell injection group had statistically significantly fewer metastatic foci than the aggregated cell injection group. The numbers of lung metastatic foci in the disaggregated AH-130 or AH-109A cell injection groups were 49.50 ± 18.98 or 20.50 ± 8.58, respectively, and those in the original AH-130 or AH-109A single cell injection groups were 39.00 ± 7.21 and 15.60 ± 6.64, respectively. Despite the different treatments, no statistical difference in the focus number was observed between the two different types of single cell suspensions of AH-130 or AH-109A cells. Therefore, the enhancing effect of the protease treatment *in vitro* on the lung-colonising ability must have been a result of tumour cell aggregation *per se*. The DNase I treatment reversed both the aggregation and the increased metastatic ability of the cells.

*Effects of protease and nuclease treatments* in vivo *on tumour cell metastasis*

Untreated AH-109A cells were intravenously injected and thereafter (3 min later) α-chymotrypsin (5 mg or 10 mg per 100 g body weight of rat) was injected into tail vein to promote cell aggregation *in vivo*. The amount of α-chymotrypsin injected into the rat was determined by an enzymatic assay *in vitro* for the α-chymotrypsin inhibitory capacity of rat plasma. On the assumption that blood volume is 1/13 of the body weight and haematocrit is 40%, a dose of

5 mg α-chymotrypsin per 100 g body weight was the minimum requirement to exceed the inhibitory capacity of circulating blood. As shown in Table IV, the groups injected with α-chymotrypsin (5 mg or 10 mg per 100 g body weight) developed more pulmonary metastases (1.8 or 2.5 times more foci, respectively) than the PBS injection group. The differences in the focus number between the former two groups and the latter were statistically significant with

**Figure 3** Effects of chymostatin and EDTA on AH-130 tumour cell aggregation induced by α-chymotrypsin, and of EDTA and actin on the tumour cell disaggregation induced by DNase I. The experimental conditions were basically the same as Figure 2. Trace A, addition of α-chymotrypsin (arrow) did not cause tumour cell aggregation in the presence of chymostatin (2.5 μM). Trace B, after treatment of the cells with EDTA (5 or 10 mM, arrow with a) with addition of α-chymotrypsin (arrow with b) still induced tumour cell aggregation to the same extent as the positive control. Trace C, addition of DNase I (15 U $ml^{-1}$, arrow c) to the cells pretreated and aggregated with α-chymotrypsin (arrow a) immediately caused a decrease in light transmission indicating the occurrence of disaggregation. Trace D, after treatment with 5 mM EDTA or 10 μg $ml^{-1}$ of actin (arrow b) of the cell aggregates induced by α-chymotrypsin (arrow a), the addition of DNase I (arrow c) did not cause the disaggregation.

**Table II** Effect of protease-induced tumour cell aggregation on pulmonary metastases in Donryu rats

| *Cell line and treatment* | *No. of metastatic foci in the lung* | | |
|---|---|---|---|
| | *Mean ± s.d* | *Range* | *P* |
| AH-130 | | | |
| α-chymotrypsin | 71.10 ± 16.68 | 40–98 | <0.01 |
| PBS | 42.40 ± 11.50 | 26–61 | |
| AH-109A | | | |
| α-chymotrypsin | 38.50 ± 16.08 | 13–66 | <0.01 |
| PBS | 17.20 ± 8.30 | 4–30 | |

Tumour cells (1 × $10^6$) were intravenously inoculated into the tail vein of Donryu rats ($n$ = 10 in each group). The aggregated cell groups received an intravenous injection of tumour cell suspension pretreated with α-chymotrypsin (100 μg per 0.1 ml). The original single cell group was pretreated with PBS. On day 12 after inoculation, the metastatic foci on the lung surface were counted.

$P<0.01$. These results indicated that the aggregation-associated enhancement of blood-borne metastasis could also be produced *in vivo* by systemic treatment with the protease. The reversing effect of DNase I in experiments with systemic treatment was then examined. In this case, α-chymotrypsin (5 mg per 100 g body weight) was injected 3 min after tumour cell inoculation and DNase I (1.5 U per 0.1 ml) was injected after a further 3 min. The effect of such systemic DNase I treatment on the number of lung foci resulting from injection of single tumour cells was also examined, i.e. untreated AH-109A cells were intravenously injected and, 3 min later, DNase I (0.3 U per 0.1 ml) was injected into the contralateral tail vein. As shown in Table V, the augmentation of lung metastasis caused by the α-chymotrypsin injection was reversed by the injection of DNase I following α-chymotrypsin. In addition, injection of DNase I alone produced a 37% reduction in the number of metastatic foci as compared with the PBS injected group. The difference in the focus number was statistically significant ($P<0.05$). The results therefore indicate that DNase I is able to reduce the number of lung foci produced by intravenously injected tumour cells.

### Discussion

The intravenous injection of protease-induced tumour cell aggregates caused more pulmonary metastases than an equal number of single cells. The higher metastatic potency was reversed when the cell aggregates were redispersed into single cells by treatment with DNase I. Therefore, it seems likely that the aggregated state itself, rather than some other possible change caused by the protease treatment, is responsible for the increased number of metastases. Emboli formation has been postulated to be advantageous for circulating tumour cells to arrest in distant organs and subsequently develop into metastatic foci. Fidler (1973) originally demonstrated the importance of cluster formation of circulating tumour cells in metastasis using B16 melanoma cells in mice. The results of the present experiments may relate to emboli formation in the pulmonary circulation. Injection of single cells followed by α-chymotrypsin was an attempt to promote protease-induced tumour cell aggregation in the blood stream. Although there is no evidence for the actual occurrence of such aggregation, the number of metastatic foci which resulted was similar to that caused by the injection of preformed aggregates. A dose of 5 mg per 100 g body weight of α-chymotrypsin was the minimum requirement to exceed the α-chymotrypsin neutralising capacity calculated for whole blood. A lower dose of α-chymotrypsin (such as 100 μg per 100 g body weight) did not cause enhancement of metastasis (data not shown). Therefore, it seems likely that the enhancement of metastasis was due to the enzymatic activity of α-chymotrypsin injected. The dose dependent enhancement of the metastasis between 5 and 10 mg per 100 g body weight of α-chymotrypsin also supports this concept. Although

**Table III** Reversibility of the enhancing effect of protease-induced aggregation on pulmonary metastases

| *Cell line and treatment* | *No. of metastatic foci in the lung* | | |
|---|---|---|---|
| | *Mean ± s.d* | *Range* | *P* |
| AH-130 | | | |
| α-chymotrypsin and DNase I | 49.50 ± 18.98 | 28–98 | <0.01 (vs α-chymotrypsin) |
| α-chymotrypsin | 85.80 ± 21.26 | 50–120 | |
| PBS | 39.00 ± 7.21 | 28–50 | n.s. (vs α-chymotrypsin and DNase I) |
| AH-109A | | | |
| α-chymotrypsin and DNase I | 20.50 ± 8.58 | 5–32 | <0.01 (vs α-chymotrypsin) |
| α-chymotrypsin | 42.70 ± 13.47 | 20–64 | |
| PBS | 15.60 ± 6.64 | 6–26 | n.s. (vs α-chymotrypsin and DNase I) |

Tumour cells ($1 \times 10^6$) were intravenously inoculated into the tail vein of Donryu rats ($n = 10$ in each group). The disaggregated cell group was successively treated with α-chymotrypsin (100 μg per 0.1 ml) and DNase I (1.5 U per 0.1 ml). The aggregated cell group was treated with α-chymotrypsin (100 μg per 0.1 ml). The original single cell group was treated with PBS. On day 12, the metastatic foci on the lung surface were counted. n.s. = no signficant difference.

**Table IV** Enhancement of pulmonary metastases in single tumour cell inoculated Donryu rats by systemic treatment with protease

| *Experiments* | *No. of metastatic foci in the lung* | | |
|---|---|---|---|
| | *Mean ± s.d.* | *Range* | *P* |
| PBS | 13.40 ± 5.17 | 6–22 | <0.01 (vs 10 mg); <0.01 (vs 5 mg) |
| α-chymotrypsin 10 mg | 33.50 ± 11.52 | 18–52 | |
| 5 mg | 24.50 ± 5.80 | 15–33 | |

Untreated AH-109A tumour cells ($1 \times 10^6$) were intravenously inoculated into the tail vein of Donryu rats ($n = 10$ in each group). Three min later α-chymotrypsin (10 mg or 5 mg per 100 g body weight) was injected into the contralateral tail vein. 0.1 ml of PBS was injected 3 min after tumour cell inoculation in the control group. On day 12 after inoculation, the metastatic foci on the lung surface were counted.

**Table V** Effects of systemic DNase I treatment in reducing pulmonary metastases of rat ascites tumour cells

| *Experiments* | *No. of metastatic foci in the lung* | | |
|---|---|---|---|
| | *Mean ± s.d.* | *Range* | *P* |
| α-chymotrypsin | 39.50 ± 11.54 | 23–58 | <0.01 |
| α-chymotrypsin and DNase I | 21.75 ± 8.89 | 8–34 | |
| PBS | 23.38 ± 6.70 | 12–32 | <0.05 |
| DNase I | 14.75 ± 6.34 | 2–22 | |

Untreated AH-109A tumour cells ($1 \times 10^6$) were intravenously injected into a tail vein of Donryu rats ($n = 8$ in each group). α-Chymotrypsin (5 mg per 100 g body weight) and DNase I (1.5 U per 0.1 ml) were injected in this order at 3 min intervals after the tumour cell inoculation. In the positive control group, α-chymotrypsin (5 mg per 100 g body weight) alone was injected 3 min after the tumour cell inoculation. In another group, DNase I (0.3 U per 0.1 ml) was injected alone into the other tail vein 3 min after the tumour cell inoculation. In the control group, 0.1 ml of PBS was injected after the tumour cell inoculation. On day 12 after inoculation, the metastatic foci on the lung surface were counted.

systemic treatment with α-chymotrypsin was used in the present model experiments, locally generated proteases in blood vessels must be the real candidate agent causing unfavourable tumour cell aggregation during clinical metastasis. The fact that anti-inflammatory agents and neutral proteinase inhibitors prevented pulmonary metastasis in mice bearing Lewis lung carcinoma (Giraldi *et al.*, 1980) would appear to support our speculation.

In our *in vitro* experiments, aggregated cells were redispersed by treatment with DNase, indicating that DNA molecules must be essential for keeping the cells aggregated. However, treatment of a single cell suspension with exogenous thymic DNA did not cause cell aggregation (data not shown). Therefore, the essential molecule is not DNA alone but a complex between DNA and some other molecule. The native of such a DNA complex is an interesting and important question to be answered. There is a speculation that nucleoprotein might be released from ruptured dead cells by protease treatment (Steinberg, 1963), and Fidler (1970) has emphasised an important role for dead tumour cells in the formation of clinical metastases. In the present experiments, however, no difference in the viability of tumour cells was observed before and after the enzyme treatment, at least as determined by the LDH assay or by the dye exclusion test. Therefore, we did not obtain any evidence for the speculation that the DNA complex is chromosomal in origin. Alternatively, Rosenberg's group has pointed to the presence of DNA on the cell surface and a correlation between the amount of surface DNA and the metastatic potency of tumour cell lines (Aggarwal *et al.*, 1975). To examine this possibility, we carried out an experiment in which the tumour cells were initially treated with DNase I to hydrolyse any such surface DNA. However, when these cells were treated with α-chymotrypsin after extensive washing to remove DNase I, the cells were able to aggregate to the same extent as the DNase I untreated control cells (data not shown), suggesting that the DNA complex is not simply present on the cell surface. Hence, in order to reach conclusions about the origin of the DNA involved in the tumour cell aggregation, as presented here, further detailed work is required in future. Nevertheless, it is of interest that systemic DNase I treatment reduced even the base-line level of pulmonary metastases in the present study (Table V). There has been a previous exciting report of reduction in leukaemic cell metastasis in AKR mice treated with repetitive intraperitoneal DNase injection (Salganik *et al.*, 1967).

In the present study, we were not able to elucidate the mechanism involved in the tumour cell aggregation brought about by protease treatment. The proteases may modify either the DNA complex (if it is a nucleoprotein) or protein moieties on the tumour cell membrane. However, even in the former case, the mechanism must be complicated, since the DNA complex does not seem to be simply present on the cell membrane.

In the literature, Weiss (1958) first reported that trypsin induced Sarcoma 37 ascites cell aggregation over 30 years ago. A similar phenomenon seems to have been observed in the classical research in experimental embryology in the 1950s (Auerbach & Grobstein, 1958; Rinaldini, 1958; Moscona, 1961; Steinberg, 1963). Auerbach and Grobstein (1958) reported the appearance of 'gummy material' which trapped single cells and small cell masses when they attempted to prepare free cells from kidney rudiment by treatment with trypsin or chymotrypsin. They also mentioned that these aggregates disappeared in a crude DNase solution. These phenomena, observed by the ontogenists and ourselves, might therefore be caused by a similar mechanism.

The authors are grateful to Dr Ishimaru of the Department of Pathology, Kumamoto University Medical School Hospital, for supplying the rat ascites tumour cells. We also thank Bro. Patrick for his kindness in revision of the English in the manuscript.

### References

AGGARWAL, S.K., WAGNER, R.W., MCALLISTER, P.K. & ROSENBERG, B. (1975). Cell-surface-associated nucleic acid in tumorigenic cells made visible with platinum-pyrimidine complexes by electron microscopy. *Proc. Natl Acad. Sci. USA*, **72**, 928.

AUERBACH, R. & GROBSTEIN, C. (1958). Inductive interaction of embryonic tissues after dissociation and reaggregation. *Exp. Cell Res.*, **15**, 384.

BOSMANN, H.B., BIEBER, G.F., BROWN, A.E. & 4 others. (1973). Biochemical parameters correlated with tumor cell implantation. *Nature*, **246**, 487.

CABAUD, P.G. & WRÓBLEWSKI, F. (1958). Colorimetric measurement of lactic dehydrogenase activity of body fluids. *Am. J. Clin. Pathol.*, **30**, 234.

FIDLER, I.J. (1970). Metastasis; quantitative analysis of distribution and fate of tumor emboli labelled with $^{125}$I-5-iodo-2′-deoxyuridine. *J. Natl Cancer Inst.*, **45**, 773.

FIDLER, I.J. (1973). The relationship of emboli homogeneity, number, size and viability on the incidence of experimental metastasis. *Eur. J. Cancer*, **9**, 223.

GIRALDI, T., SAVA, G., KOPITAR, M., BRZIN, J. & TURK, V. (1980). Neutral proteinase inhibitors and antimetastatic effects in mice. *Eur. J. Cancer*, **16**, 449.

HARA, Y., STEINER, M. & BALDINI, M.G. (1980). Characterization of the platelet-aggregating activity of tumor cells. *Cancer Res.*, **40**, 1217.

KINJO, M. (1978). Lodgement and extravasation of tumour cells in blood-borne metastasis. An electron microscope study. *Br. J. Cancer*, **38**, 293.

LIOTTA, L.A., TRYGGVASON, K., GARBISA, S., HART, I., FOLTZ, C.M. & SHAFIE, S. (1980). Metastatic potential correlates with enzymatic degradation of basement membrane collagen. *Nature*, **284**, 67.

MOSCONA, A. (1961). Rotation-mediated histogenetic aggregation of dissociated cells. *Exp. Cell Res.*, **22**, 455.

NICOLSON, G.L. & WINKELHAKE, J.L. (1975). Organ specificity of blood-borne tumour metastasis determined by cell adhesion? *Nature*, **255**, 230.

RINALDINI, L.M. (1958). The isolation of living cells from animal tissues. *Int. Rev. Cytol.*, **7**, 587.

SALGANIK, R.I., MARTYNOVA, R.P., MATIENKO, N.A. & RONICHEVSKAYA, G.M. (1967). Effect of deoxyribonuclease on the course of lymphatic leukaemia in AKR mice. *Nature*, **214**, 100.

SLOANE, B.F., HONN, K.V., SADLER, J.G., TURNER, W.A., KIMPSON, J.J. & TAYLOR, J.D. (1982). Cathepsin B activity in B16 melanoma cells: a possible marker for metastatic potential. *Cancer Res.*, **42**, 980.

STEINBERG, M.S. (1963). 'ECM': its nature, origin and function in cell aggregation. *Exp. Cell Res.*, **30**, 257.

WANG, B.S., MCLOUGHLIN, G.A., RICHIE, J.P. & MANNICK, J.A. (1980). Correlation of the production of plasminogen activator with tumor metastasis in B16 mouse melanoma cell lines. *Cancer Res.*, **40**, 288.

WEISS, L. (1958). The effects of trypsin on the size, viability and dry mass of Sarcoma 37 cells. *Exp. Cell Res.*, **14**, 80.

WEXLER, H. (1966). Accurate identification of experimental pulmonary metastasis. *J. Natl Cancer Inst.*, **36**, 641.